# A Review of Telehealth Service Implementation Frameworks

**DOI:** 10.3390/ijerph110201279

**Published:** 2014-01-23

**Authors:** Liezl van Dyk

**Affiliations:** Industrial Engineering, Faculty of Engineering, North-West University, Potchefstroom Campus, Potchefstroom 2520, South Africa; E-Mail: liezl.vandyk@nwu.ac.za; Tel.: +27-18-299-1524; Fax: +27-18-299-1320

**Keywords:** telehealth, telemedicine, implementation, framework, model

## Abstract

Despite the potential of telehealth services to increase the quality and accessibility of healthcare, the success rate of such services has been disappointing. The purpose of this paper is to find and compare existing frameworks for the implementation of telehealth services that can contribute to the success rate of future endeavors. After a thorough discussion of these frameworks, this paper outlines the development methodologies in terms of theoretical background, methodology and validation. Finally, the common themes and formats are identified for consideration in future implementation. It was confirmed that a holistic implementation approach is needed, which includes technology, organizational structures, change management, economic feasibility, societal impacts, perceptions, user-friendliness, evaluation and evidence, legislation, policy and governance. Furthermore, there is some scope for scientifically rigorous framework development and validation approaches.

## 1. Introduction

Telehealth has the potential to address diverse problems in modern healthcare by increasing the quality, accessibility, utilization, efficiency and effectiveness of healthcare, with the added advantage of cost reduction [1,2]. Despite its potential, the success rate of telehealth services has been disappointing [3,4,5]. Apart from the obvious waste of equipment and human resources, Yellowlees [2] considers the damage to the reputation of telehealth an even greater expense. The problem is, firstly, that many telehealth services, which proved to be successful in the pilot phase, could not be sustained. Secondly, and an even a greater obstacle, is that many mistakes in the implementation are repeated over and over again, while only a few examples of good practices are replicated. 

The complexity of a telehealth service is often underestimated. There are numerous factors that have an impact on its success, ranging from technological issues to infrastructure, legislation, change management and financial business models. Telehealth services, by definition, are delivered over a distance and, thus, always span more than one organizational entity. These entities often exhibit conflicting organizational cultures and practices, as well as incompatible business models and governing processes. Furthermore, telehealth services involve multidisciplinary role players, ranging from a wide variety of healthcare workers and information and communication technologists, to economists, managers and policy makers. The way in which decisions are executed, problems solved and change managed is also often linked to a specific discipline, which adds to the complications emanating from the implementation of telehealth services. 

## 2. Purpose

Persons responsible for the implementation of telehealth services are seeking for frameworks as a guide to their effective operationalization. The purpose of this paper is, therefore, to find and compare existing frameworks for the implementation of telehealth services in order to identify common themes and formats, as well as to identify areas for future development. The research questions are as follows:

(1) What are the common themes and formats used in these frameworks?
(2) Which methodologies were followed to develop these frameworks? On which theories are they based? Which research methods were used to develop these frameworks? How are they validated? What are the areas for further research?

## 3. Methodology

The literature search included peer-reviewed articles and chapters from books found in academic databases. These are indicated in the column headings used in Table 1. The next section considers the definition of *telehealth*, *telemedicine* and related concepts, thereby providing the rationale for including both *telehealth* and *telemedicine* as search terms. These are listed in the row headings of Table 1. 

The number of articles found in each search is also indicated in this table. A total of 491 papers were found, with a few papers common to more than one search. In each case, the abstract was read to identify those that could possibly contain an actual model, framework or set of guidelines useful for the implementation of a telehealth service. These were then scrutinized based on the content of the entire paper. 

Individual case study reports were excluded, unless they contained frameworks that were applicable to telehealth services beyond the context of a particular study. Systematic reviews were excluded [6,7], unless they culminated in an actual model, framework or list of guidelines [3]. Frameworks that were directed to a specific telehealth application, for example Picture Archiving and Communication Systems (PACS) [8], were only included if they do not exhibit application-specific technical and implementation detail and, hence, could be applied to the implementation of a telehealth service beyond this context. 

**Table 1:** Search terms and databases.

Search terms and database	Science Direct	IEEE	EBSCO Host	Scopus	ProQuest	PubMed
(telemedicine OR telehealth) AND implementation AND framework	14	19	13	81	6	81
(telemedicine OR telehealth) AND implementation AND model	29	43	27	200	18	159
(telemedicine OR telehealth) AND implementation AND guidelines	4	5	8	62	4	59

Although evaluation and implementation go hand in hand, evaluation does not necessarily contribute to successful implementation. However, in this paper, some evaluation frameworks are included, not only because they serve as a means of evaluation, but also because they are a guide for implementation. Articles, such as *A Framework for the Economic Evaluation of Telemedicine* [9] and the *Model for the Assessment of Telemedicine (MAST)* [10] were excluded, because their focus was primarily on the evaluation of the outcomes, and they did not provide guidelines on the implementation of the service. After completion of this process, 9 papers were selected for review in this study. Nonetheless, much of the excluded material provided relevant background information and will therefore be referred to in this paper. Table 2 shows a summary of the relevant development methodologies and common themes in the frameworks found in the literature. 

This paper is structured as follows: *Telehealth* and related concepts are firstly discussed, so as to specify the scope for this paper, as well as to provide a rationale for using *telemedicine* and *telehealth* as search terms. This is followed by a brief description of each of the 9 frameworks. For the purposes of this discussion, frameworks with similar underlying theories, development approaches and framework formats are grouped together:

**Section 5:** Frameworks related to the *diffusion of telemedicine*
**Section 6:** Frameworks related to *ereadiness*
**Section 7:** Telehealth applications of the *Unified Theory for the Acceptance of Technology*
**Section 8:** Guidelines that are not based on a particular theory, but retrospectively on the implementation of some telehealth services
**Section 9:** The Comprehensive Model for the Evaluation of Telemedicine [11]
**Section 10:** Frameworks that incorporate the lifecycle phases of a telehealth service

**Table 2:** Comparison of frameworks

	Framework	Underlying Theory	Research and Development Methods	Validation Methods	Framework Format	Main Themes
5	Barriers to the diffusion of telemedicine	Diffusion of Innovation [12]	A longitudinal study of three telehealth programs.	No formal validation	Four so-called barriers diffusion of telehealth services.	technological, organizational, behavioral and economic barriers
6	eHealth readiness assessment tools	Theories on ereadiness and change management	Adaption of existing ereadiness scales based on input from ehealth experts.	Expert interviews, as well as statistical reliability testing of questionnaire results [13]	A set of questionnaires, including 51 statements linked to a Likert scale.	technology, learning, society, economical, policy
7	Unified Theory of Acceptance and Use of Technology (UTAUT) applied to telehealth [14,15]	Unified Theory of Acceptance and Use of Technology (UTAUT)	UTAUT questionnaire administered with specific reference to telehealth.	UTAUT questionnaire validated through other studies; telehealth application validate by experts.	A list of statements linked to a Likert scale.	technology perceived usefulness, perceived ease of use, behavioral intent, demographic factors
8.1	Seven Core Principles for the Successful Development of Telemedicine Systems	No particular theory	Literature and personal experience of setting up three telehealth services in Australia.	No formal validation.	Guidelines structured according to seven principles.	ownership, bottom-up support, user-friendliness of technology, training, dissemination of evidence
8.2	Lessons in telemedicine service innovation	No particular theory	Longitudinal qualitative study; data gathered by means of questionnaires.	No formal validation	Guidelines structured according to 5 lessons.	policy, evidence, perceived benefit, commitment, service design, professional roles and border crossing
8.3	Framework for Assessing the Health System Challenges to Scaling up mHealth	ICT for health in developing countries Khoja *et al.* [16] Bukachi and Pakenham-Walsh [17]	Qualitative study, combination of reviews with key informants, site visits to local projects and documented reviews.	No formal validation	Four dimensions, each with a collection of capatiy requirements.	government, organization, technology, finances.
9	Comprehensive Model for the Evaluation of Telemedicine	Theories of Transactional Economics [18]	Design and integration of three evaluation dimensions.	No formal validation	A three-dimensional framework; each dimension has several categories.	individual, community, society, cost, quality, access.
10.1	The Layered Telemedicine Implementation Model	Knowledge barriers to the diffusion of telemedicine Tanriverdi and Iacono [19]	Systematic literature review of 45 articles on the implementation of telemedicine services.	No formal validation	5 lifecycle phases, each of a collection of determinants for success.	technology, acceptance, organization, policy and legislation
10.2	The Khoja– Durrani–Scott (KDS) Evaluation Framework	Concepts and theories related to the evaluation of ehealth (no mention of which) as well as system lifecycle theories	Systematic literature review and expert opinions.	Validation to be published in another paper	A list of desired outcomes per lifecycle phase per theme; each outcome linked to a Likert-like scale.	evidence, technology, economic, behavioral and sociotechnical, thical, change management, policy

## 4. eHealth, Telehealth, Telemedicine, Telecare and mHealth

**Figure 1:**
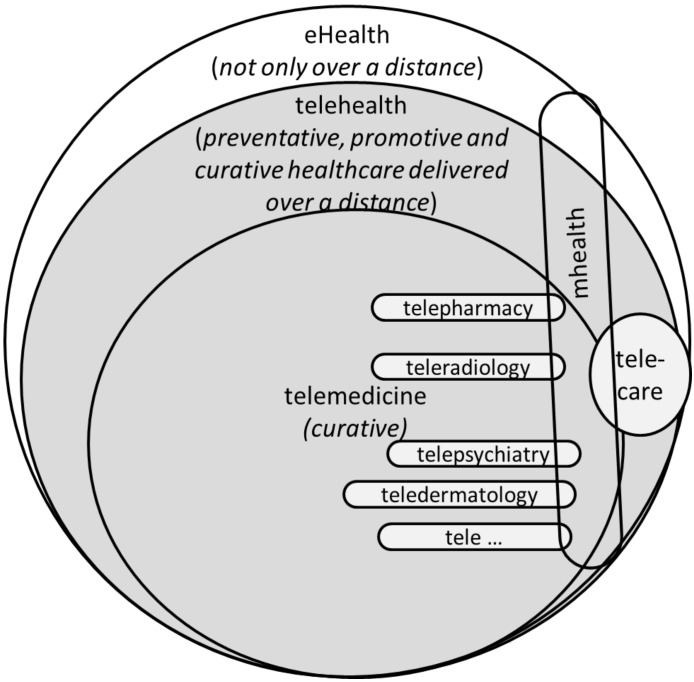
Telemedicine, ehealth, telehealth, telecare and mhealth.

Figure 1 shows the relation between ehealth, telehealth, telemedicine, telecare and mhealth, as discussed in this section. The grey area indicates the range of telehealth services, which is also the scope of this paper. Only the frameworks that are related to healthcare services spanning a distance (tele-) were included. 

### 4.1. Telehealth

Bashshur *et al.* [20] explain that telehealth relates to telemedicine the same way that health relates to medicine. Sood *et al.* [6], after considering 104 peer-reviewed definitions for telemedicine, concluded that telemedicine is a subset of telehealth. According to Bashshur *et al.* [20], Bennet *et al.* coined the term *telehealth* in 1978 to extend the scope of telemedicine by incorporating a “broader set of activities, including patient and provider education.” 

The notion that telemedicine is a subset of telehealth [6,20] is reinforced by the community who maintain the telehealth wiki page. They see telehealth as an expansion of telemedicine, but unlike telemedicine, which has a narrower focus on the curative aspect, it encompasses the preventative, promotive, as well as the curative aspects of the field. 

### 4.2. Telecare

According to the Telecare Aware Group [21], “telecare is the continuous, automatic and remote monitoring of real time emergencies and lifestyle changes over time in order to manage the risks associated with independent living.” As a preventative health application, it is thus within the scope of telehealth, but not telemedicine. 

*4.3. eHealth*

The terms *ehealth* and *telehealth* are most often used interchangeably. Semantically, the difference between these two concepts is that ehealth applications are not limited to healthcare over a distance, as is the case with telehealth. This distinction is maintained in this study. 

*4.4. mHealth*

*mHealth*, as a concept, appeared relatively recently in the literature on ehealth. mHealth refers to ehealth applications that are executed with the help of mobile technology. In the Telemedicine Hype Cycle Report by the Gartner Group, Handler [5] is critical of what he calls the “over-excitement” surrounding mhealth. He considers the term to be obsolete, because mobile technologies are now routinely incorporated into the delivery of healthcare. Bashshur *et al.* [20] also draws attention to the fact that mhealth is the only ICT-based health domain that is solely justified on the basis of mobility and related technology. Despite their misgivings that mhealth is conceptually and empirically differentiated from telemedicine, they have incorporated it into their taxonomies of telemedicine, due to the widespread use of the term. 

For the purposes of this study, mhealth is considered to be a subcategory of ehealth, telehealth and telemedicine and cuts across these categories, as is shown in Figure 1. The emphasis is on the means (mobile technologies) and not necessarily the end (healthcare delivery) [5]. 

## 5. Frameworks Related to the Diffusion of Innovation

Over the past few decades, researchers have been increasingly intrigued by innovation and the factors that impact on its diffusion. This interest has intensified as technology and technological innovations have developed and the complexity of enterprises has increased. The barriers to the diffusion of telemedicine [19] and the influences on the diffusion of telemedicine [22] have been responsible for delivering some important concepts to the telehealth research domain. 

Rogers’ categories of innovation adoption, namely innovators, early adopters, early majority, late minority and laggards, can be recognized in the diffusion of telehealth innovation. The many pioneering initiatives of innovators [5,9] occurred during the first few decades after the term *telehealth* was used for the first time (1970). Then, from about 1995, motivated by significant ICT developments [1,22], telehealth applications were used not only by pioneers and innovators, but also by early adopters who hoped to improve their daily work in healthcare. Today, telehealth is becoming a key issue in the implementation of healthcare services and is of interest to the early majority. 

Scott [23] brought Rogers’ Diffusion of Innovation Theory into the healthcare domain by applying it to medical care organizations. Grigsby *et al.* [22] then narrowed the focus to telehealth services and compiled a list of factors that influence the adoption of innovative technologies, based on the work by Scott [23]. 

Tanriverdi and Iacono [19] were the first to recognize that insights concerning the diffusion of innovation can be applied to the implementation of telehealth services. They based their research on the work of Attewell [12] concerning technology diffusion and organizational learning. He defined four so-called innovation barriers. Tanriverdi and Iacono [19] translated these into barriers for the diffusion of telehealth. These barriers and some of the observations by Tanriverdi and Iacono [19] with respect to telehealth are listed below:

**Technical barrier:** It is imperative that the appropriate technology is available, as well as knowledge about it.
**Behavioral barrier:** This barrier involves change management, especially with respect to resistance to change, power and politics around telehealth. Tanriverdi and Iacono [19] emphasized the importance of so-called *proponents of telehealth* in accomplishing this change.
**Economical barrier:** Two major concerns of Tanriverdi and Iacono [19] were to reimburse healthcare workers for telehealth consultations and to open up new patient markets.
**Organizational barrier:** Tanriverdi and Iacono [19] found it crucial to integrate telehealth services into existing organizational structures and to provide institutional support to execute these services.

## 6. eReadiness Frameworks

Theories on the adoption and diffusion of innovation, as well as Lewin’s Three Phase Model [24] form the basis for theories on readiness. eHealth and telehealth readiness is defined as the degree to which a community is prepared to participate and succeed in the ehealth or telehealth service and is normally measured before the implementation of the service [17]. It considers both the capacity for making changes, as well as the perceived need to change. Jennett *et al.* [16] specifically refer to ehealth readiness when arguing that time, money and energy can be saved if the status quo of an ehealth/telehealth system context is determined before implementation. 

A few readiness instruments have already been developed and are in use within the context of telehealth and ehealth. Legare *et al.* [25] identified six different assessment tools that use Likert scale questionnaires to measure e-readiness within a certain healthcare context. The first of these tools was developed in 1996. The Organizational Information Technology/Systems Innovation Readiness Scale supports the evaluation, diagnosis and treatment selection for different steps in patient care, within the domain of telehealth. 

The second, third and fourth set of tools mentioned by Legare *et al.* [25] were built upon each other and are focused on home-based telehealth applications. Khoja *et al.* [16] developed the eHealth Readiness Assessment Tool Set for Healthcare Institutions in Developing Countries and Jennett *et al.* [24] developed a telehealth readiness assessment tool that focuses on ehealth applications in rural settings. 

The tool set by Khoja *et al.* [16] is significant for two reasons. It firstly forms the basis of a recent evaluation framework [26], which is considered in the next section. Secondly, this tool set has had the most favorable reception by the academic community, if measured by the rate at which it is cited by others. Several authors have used this tool set as a reference in the development of other telehealth and ehealth assessment frameworks [27,28,29]. The following are two publications that have specifically used this tool set in their ehealth readiness studies:

**Chipps and Mars** [30] assessed the preparedness of health districts and designated hospitals in the KwaZulu-Natal (KZN) province for proposed telepsychiatry services. They concluded that in order for telepsychiatry to succeed in KwaZulu-Natal, a change management awareness would be needed. However, it was not made clear if and how the ehealth readiness assessment tool set could assist with this.
**Durrani and Khoja** [31] used this tool set to measure the ehealth readiness of two separate ehealth programs, one in Kabul and the other in Bamyan. The ehealth readiness assessment tool set was found to be useful, firstly, in comparing the ehealth readiness of these two programs, and secondly, in “broadening the vision of the institutions as a whole”.

The following set of ehealth readiness assessment tools for healthcare institutions in developing countries [16] covers five categories. Each category contains a number of statements, which a respondent is asked to agree/disagree with, according to a five-point Likert scale. Each of these statements addresses a single determinant of access to ehealth. The way in which the statements are expressed, together with the Likert scale, provides a means of quantifying the perceived ehealth readiness.

(1) **Core readiness** (21 statements) deals with aspects of planning and integration.
(2) **Technological readiness** (10 statements) considers the availability, reliability, affordability and ICT, as well as the related infrastructure.
(3) **Learning readiness** (six statements) addresses issues related to the programs and resources available for the provision of training in the use of the technology.
(4) **Societal readiness** (11 statements) considers the interaction between the institution and other institutions in the region and beyond. Socio-cultural factors are also included.
(5) **Policy readiness** (12 statements) deals with policies, at the government and institutional level, which are in place to address common issues, such as licensing, liability and reimbursement.

Khoja *et al.* [16] grouped these sets of statements into two so-called tool sets. The first tool set is targeted at managers and includes core readiness, technological readiness, societal readiness and policy readiness, but excludes learning readiness. The second tool set, which is targeted at healthcare providers, includes core readiness, learning readiness, societal readiness and policy readiness, but not technological readiness. Table 3 is compiled to show the relationship between the work of Tanriverdi and Iacono [19] and the work of Khoja *et al.* [16]. This indicates that these questionnaires address categories by Tanriverdi and Iacono [19] a micro-, as well as a macro-level. 

**Table 3:** Relationship between Tanriverdi and Iacono [19] and Khoja *et al.* [16].

Barriers [19]	Micro-level	Macro-level
**Technical**	**Technology** (hardware and software)	**Technology** (ICT infrastructure)
**Behavioral**	**Learning** (healthcare workers)	**Society**
**Economical**	**Core** (budget)	**Policy** (reimbursement models)
**Organizational**	**Core** (process integration and prioritization)	**Policy** (planning and promotion of telehealth)

## 7. Unified Theory of Acceptance and Use of Technology (UTAUT)

The Unified Theory of Acceptance and Use of Technology (UTAUT) was the result of a study by Venkatesh *et al.* [32], who synthesized eight theories/models of technology use. Since then, many expansions and adaptations of the UTAUT have been published. The UTAUT comprises a model, which indicates the interaction between different variables that determine the acceptance of technology. This model is accompanied by a questionnaire that contains a list of statements related to each of these variables. Respondents are presented with a Likert scale, where they are asked to indicate the extent to which they agree or disagree, for example:

- I find (the technology under consideration) useful in my daily life.
- Using (the technology under consideration) helps me accomplish things more quickly.
- The (technology under consideration) is reasonably priced.
- The use of (the technology under consideration) has become a habit for me.

Dünnebeil *et al.* [33] administered their questionnaire to 117 physicians and found that the perceived importance of standardization and current IT utilization were the most significant drivers for their accepting electronic health services (EHS) in their practices. Cilliers and Flowerday [14] used the UTAUT to investigate user acceptance of telehealth in the public healthcare system in the Eastern Cape (South Africa). They concluded that “in general, the acceptance of telemedicine in the Eastern Cape Department of Health is positive, but in order to integrate it into standard work practices, more must be done with regards to the promotion and education of telehealth”. 

## 8. Retrospective Guidelines

Most frameworks in this paper are based on other theories, for example the diffusion of innovation or ereadiness or theories of technology acceptance. However, the frameworks discussed in this section are not based on a particular theory. They are lists that were compiled retrospectively, based on knowledge that was gained through the experience of developing, implementing and optimizing telehealth services. 

### 8.1. Seven Core Principles for the Successful Development of Telemedicine Services

Yellowlees [2] defined seven broad principles, based on his experience in setting up three telehealth systems in Australia. He intended these principles to be applicable to any telehealth system, whether newly developed or in operation for some time. These principles are listed below:

(1) Telehealthcare applications as sites should be selected pragmatically, rather than philosophically.
(2) Clinician drivers and telehealth users must own the systems.
(3) Telehealthcare management and support should be from the bottom up rather than from the top down.
(4) The technology should be as user-friendly as possible.
(5) Telehealthcare users must be well trained and supported, both technically and professionally.
(6) Telehealthcare applications should be evaluated in a clinically appropriate and user-friendly manner.
(7) Information about the development of telehealth must be shared.

### 8.2. Lessons in Telemedicine Service Innovation

Finch *et al.* [34] conducted a longitudinal study between 1997 and 2005 on twelve existing teledermatology services. In the course of the study, 68 interviews with service role players took place to identify the factors that contributed to these services becoming routine practice. These factors were organized into five themes:

**Policy context:** Policies should be formulated in such a way that telehealth innovation is encouraged rather than discouraged. It is also important that the policies are translated into resources.
**Evidence gathering, “proving” safety and managing risk:** The successful telemedicine services were those whose potential risks were acknowledged and for which safeguards were built into the systems. Furthermore, such services emphasized the close monitoring of effects and outcomes, rather than formal, scientific, evaluation.
**Perceived benefit and related commitment:** Finch *et al.* [34], without consulting theories on the acceptance of technology, concluded that there is a direct link between the willingness of role players to commit to new technology and/or methods and the benefit they are perceived to have.
**Reconfiguring services:** The focus should not be on the technology, but on the way in which the service is delivered.
**Professional roles and boundary crossing:** Together with changes in work procedures, clinicians need to make changes to their traditionally perceived professional roles.

### 8.3. Framework for Assessing Health System Challenges to Scaling up mHealth in South Africa

Leon *et al.* [29] published a framework that was developed after nineteen interviews with key figures in the field of mHealth, an assessment of three local mhealth projects and a review of grey and indexed literature. An adaptation of this framework is presented in Table 4. They defined four so-called dimensions, which are strongly reminiscent of the barriers to the diffusion of telemedicine by Tanriverdi and Iacono [19], although the authors did not make specific reference to these elements. For each of these dimensions, a set of so-called capacity requirements is defined. 

**Table 4:** Adaptation of the Framework for Assessing the Health System Challenges [29].

**Government stewardship:** is there a policy environment supportive of mhealth?
**Strategic leadership:** policy guidelines, alignment with strategic health goals, funding sources, common ICT standards, collaboration partnerships
**Learning environment:** learning environment, systematic evaluation of projects, central repository of projects
**Organizational:** culture of and capacity for using information technology for management
**Capacity for implementation:** capacity to implement mhealth interventions, assessment of ereadiness, a functional ICT environment and effective mechanisms for implementation, support, monitoring and evaluation.
**Culture of information use:** organizational culture of using health information for management
**Technological:** integrated and sustainable technology
**Use-ability:** ease of use, flexibility and durability, beneficial to end users
**Interoperability:** communication across technological and information platforms, integration with existing work practices, common standards, financial sustainability
**Privacy and security:** privacy and security of data, regulations for protecting electronic data
**Financial:** financial provision being made for the medium to long term
**Sustainable funding:** sustainable funding for large-scale implementation, clear business and funding plans
**Cost-effectiveness:** cost-effectiveness evaluated, mhealth interventions weighed up against other priority and evidence-based interventions; opportunity costs are routinely considered

## 9. Comprehensive Model for Evaluating Telemedicine

**Figure 2:**
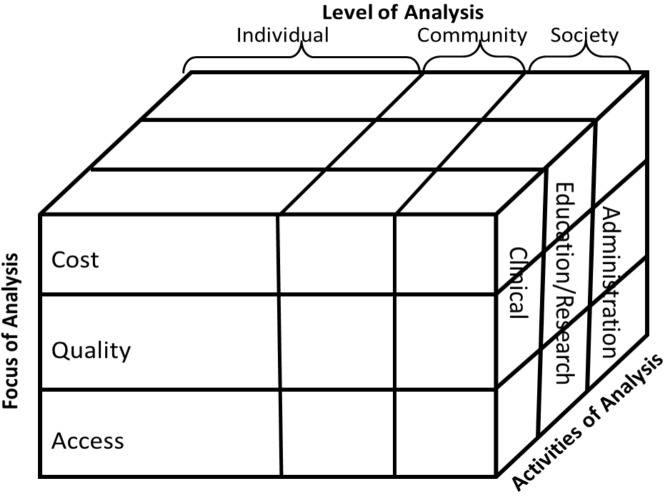
Comprehensive model for evaluating telemedicine [11].

Hicks *et al.* [11] based this model on the theories of transactional economics, also referred to as transaction cost economics, and has three dimensions, namely:

**Level of analysis:** Each of the three broad categories (individual, community and society) is comprised of multiple elements of which some examples are indicated in Figure 2. “Conclusions regarding the acceptability of telehealth may vary substantially across the three levels, since benefits and costs may accrue to entities outside the immediate transaction.” [11]
**Focus of analysis:** This dimension considers the often conflicting considerations of cost, quality and access to healthcare.
**Activities of analysis:** Telehealthcare services are mostly directed towards clinical examinations, consultations, discussions and other clinical purposes. With this dimension, it is also recognized that telehealth service infrastructure is also used for education, research and administration.

Hicks *et al.* [11] consider their model comprehensive, because their three-dimensional approach ensures that the myriad of issues related to telehealth services are all considered, for example:

- The cost of education/research concerning the service at the level of an individual;
- The level of community access to clinical services;
- The level of quality of the clinical services to society;
- An individuals level of access to administrative services;
- The quality of education/research concerning the service at the community level;
- The cost of administrative services at the society level.

It is due to the comprehensive nature of this model that it is also able to serve as a guide to the implementation of telemedicine services. Aside from its usefulness as an evaluation tool, it is effective in the identification of areas that need specific attention in the implementation of this service. 

## 10. Lifecycle Frameworks

The systems development lifecycle describes the process used in the planning, creating, testing and deploying of an information system. Similarly, a telehealth service also undergoes a number of lifecycle phases. The framework in this section links the determinants for successful implementation, as well as the expected outcomes, to a certain lifecycle phase (refer to Table 5). In doing so, it serves as a guideline for the implementation of a telehealth service throughout the lifecycle of the service. 

**Table 5:** Typical lifecycle stages for telehealth services.

Layered telemedicine implementation model [3]	Stages of the ehealth lifecycle [26]
Prototype	Development
Small-scale Pilot	Implementation
Large-scale Pilot	Integration
Operational Product	Sustained Operation

### 10.1. The Layered Telemedicine Implementation Model

Broens *et al.* [3] conducted a systematic literature review in answer to the question of why it is so difficult (to implement telemedicine services) and what goes wrong. In this study, the barriers to the diffusion of telemedicine [19] is used as a theoretical framework for the identification of the so-called determinants in the successful implementation of telehealth. Broens *et al.* [3] postulate that different determinants apply throughout the implementation lifecycle and their Layered Implementation Model was developed accordingly. The relationship between each implementation layer and its associated determinants (in brackets) is shown in Table 6. 

**Table 6:** The Layered Telemedicine Implementation Model Broens *et al.* [3].

Lifecycle Phase	Category	Determinants for the successful implementation of telemedicine
Prototype phase	Technology	support, training, usability, quality
Small-scale pilots	Acceptance	attitude and usability, evidence-based medicine, diffusion and dissemination
Large-scale pilots	Financing and organization	service provider and structure
Operational products	Policy and legislation	legislation and policy, standardization, security

During the prototype phase, the focus was on the technological feasibility of the telehealth service. According to Broens *et al.* [3], the extent to which this technology is accepted by the users and society determines the success of the pilot phase. As soon as the pilot projects were scaled up, the financial and organizational considerations determined the success of the telehealth service. Broens *et al.* [3] explain that the research stages (prototype and pilot) are most often funded externally. Many telehealth projects fail because the financial sustainability beyond the research phases was not considered. Organizational issues include the definition of standards and protocols, as well as ensuring that the organization fits the new service rather than making the technology fit the old organization. Broens *et al.* [3] describe a fully implemented service as an *operational product* and identified policy and legislation as critical to this phase. 

### 10.2. The Khoja–Durrani–Scott (KDS) Evaluation Framework

The Khoja–Durrani–Scott (KDS) Evaluation Framework (refer to Table 7) was developed as a PANACeA-initiative (PAN Asian Collaboration for Evidence-based eHealth Adoption and Application) by some of the authors who developed the ehealth readiness assessment tools. The significance of this framework lies in the fact that it acknowledges the need for different assessment strategies throughout this implementation lifecycle. This framework has several dimensions, the first two of which are presented in Table 7. The stages of the ehealth lifecycle comprise the column headings, with the themes for the evaluation lists making up the headings for the rows. 

**Table 7:** Extract from the Khoja-Durrani-Scott (KDS) Evaluation Framework [26].

Stages of the ehealth lifecycle				
Themes of Evaluation	Development	Implementation	Integration	Sustained Operation
**Health Services**	Ongoing assessment of health services status, opportunities and needs	Improved diagnosis and treatment of disease conditions	Health impact leading to change in disease status	Health impact showing change via indicators
**Technology**	Cost of development, availability, affordability	Interoperability	Appropriate in a variety of conditions	Scalability
**Economic**	Affordability	Cost-utility	Cost-utility Cost-benefit	Improved disability-adjusted life years
**Behavioral and** **Sociotechnical**	Factors related to human resources	Strategy for ehealth implementation	Strategy for broader ehealth adoption	Adoption / adaption of technology on a wider scale
**Ethical**	eHealth prioritized over other issues	Sensitive to sociocultural issues	Broader perspective on security, liability, licensure as well as reimbursement	Security
**Readiness and** **Change**	Change management planning	Training of staff, including clinical and management staff	Effective management of change	modification, improvement, customization
**Policy**	Change management policies	Limited changes in organizational and national policies	Policy changes to facilitate broader adoption	Public policy and organizational practice

For each of the four stages of the ehealth lifecycle and for each of the seven themes of evaluation (28 cells in total), Khoja *et al.* [26] defined desired outcomes. Some examples of these outcomes are indicated in Table 7. A further dimension of the KDS-framework is a collection of evaluation questionnaires. Similar to the ehealth readiness assessment tools, these questionnaires consist of a series of statements, linked to a Likert scale. While the former were scaled from not-prepared to prepared [16], the scaling of the KDS-framework is more specific: unsatisfactory, below expectations, meets expectations, above expectations and extraordinary. 

Three sets of questionnaires are available, depending on the viewpoint of the respondent. Also similar to the ehealth readiness assessment tools, a few sets of questionnaires were developed. The appropriateness set was determined by the viewpoint of the assessor, i.e., (1) manager or (2) healthcare provider. A third viewpoint category was added to the KDS, namely (3) client. 

## 11. Discussion

The purpose of this paper was to find and compare existing frameworks for the implementation of telehealth services, in order to identify common themes and formats, as well as identify areas for future development. Nine such frameworks were identified as part of a systematic review process and are described in this paper. These frameworks are summarized at the beginning of this paper (Table 2) in terms of theories on which these frameworks are built, methods used to develop and validate the framework, the format of the framework and common themes. 

### 11.1. Theories

In some cases, theoretical frameworks from other domains are adopted to telehealth services, for example theories on the diffusion of innovation, technology acceptance, ereadiness and system lifecycles. A telehealth service is an innovation that relies on the extent to which the technology is accepted by its users. In addition, it is dependent upon the readiness of the organizational systems, and like any other system, it goes through a number or lifecycle stages and should be managed accordingly. 

None of the articles reviewed in this study explained the rationale for selecting a particular theoretical framework. Although the merit of the selected theories is not in dispute, a critical analysis of existing theories from other domains related to telehealth may yield additional research artifacts applicable to the implementation of telehealth services. 

### 11.2. Development Methods and Framework Formats

The frameworks can be grouped together as follows in terms of the development method and framework formats:

**Statements/outcomes associated with Likert-like scales:** The ehealth readiness assessment tools [16] as well as the UTAUT, as it is applied to telehealth [14,15], are framed as a list of statements that is linked to a Likert-like scale, ranging from *strongly disagree* to *strongly agree*. Both of these tool sets are taken from other domains in which they were already validated.
**Guidelines based on longitudinal studies:** The barriers to the diffusion of telemedicine in [19], the Seven Core Principles in [2] and the lessons in telemedicine service innovation in [34] are all based on longitudinal studies, and they are formatted as a list of guidelines. The framework for assessing health system challenges to scaling up mhealth [29] is also included here, since it relies on case studies and consists of a list of so-called capability requirements.
**Lifecycle frameworks:** For both the KDS Evaluation Framework [26] and the Layered Implementation Model [3], systematic literature reviews were executed. Khoja *et al.* [26] also conducted interviews. Both of these are framed along the lifecycle phases of a telehealth service. Within their frame, Broens *et al.* [3] indicate, what they call, determinants for the successful implementation of telemedicine services. Khoja *et al.* [26] fill their framework with expected outcomes.
**The Comprehensive Model for the Evaluation of Telemedicine** has a unique three-dimensional design. It is not clear which method was followed to arrive at this design.

### 11.3. Validation

The validity and reliability of ehealth readiness assessment tools are tested in other papers [13]; the validity of UTAUT was confirmed even before Cilliers and Flowerday [14] and Dünnebeil *et al.* [33] applied it to telehealth. Finch *et al.* [34], Leon *et al.* [29], as well as Khoja *et al.* [26] all indicated that they had involved telehealth experts to determine whether their conclusions were valid. None of the other papers indicated an attempt at proving validation. All the frameworks have been used in follow-up studies to guide implementation, thus future studies may be able to draw upon such applications to consider the effectiveness and validity of these frameworks. 

### 11.4. Themes

Some frameworks are based on existing theories (top-down development approach). Others are based on experience (bottom-up development approach) rather than other theories. However, there is significant overlap among the themes that were distilled from both the bottom-up and top-down approaches. For example, many of the lessons by Finch *et al.* [34] can be traced back to the UTAUT, whilst the five categories of Khoja *et al.* [16] and the four dimensions defined by Leon *et al.* [29] correlate with the work of Tanriverdi and Iacono [19]. 

The themes listed in the last column of Table 2 are those themes that are particularly featured in that specific framework. The following themes were identied (the amount of frameworks that addresses each particular theme is indicated in parentheses): technology (eight); change management and organizational behavior and learning (seven); economics, finances and costs (five); policy, governance and legislation (five); organizational design and service design (four); community and society (three); evidence (three); perceived benefit and technology acceptance (three); access (two); quality (one). 

## 12. Conclusions

Despite the potential of telehealth services to increase the quality and accessibility of healthcare services, the implementation success rate of such services are disappointing. Many persons involved in the implementation of telehealth service have published guidelines and frameworks that can enable others to gain from their experience. For the purposes of this paper, nine publications were reviewed in which such frameworks and guidelines are published. It is unfortunate that only two of these articles [14,29] are available on open access platforms, since these frameworks are needed by entities who do not necessarily have access to the academic databases. 

This review confirmed, firstly, that a holistic implementation approach is needed, which includes technology, organizational structures, change management, economic feasibility, societal impacts, perceptions, user-friendliness, evaluation and evidence, legislation, policy and governance. Secondly, existing theories were identified that are developed and validated in other contexts and that can assist in the implementation of telehealth services. Possibly more such theories can be found if purposely pursued. Thirdly, some scope exists for scientifically rigourous framework development and validation approaches. 

Best-practice implementation approaches will help to unleash the potential of telehealth to address diverse problems in modern healthcare. In this paper, existing frameworks for the implementation of telehealth services are reviewed and some direction for future work is provided. 

## References

[1] Bashshur R., Reardon T., Shannon G. (2000). Telemedicine: A new health care delivery system. Annu. Rev. Public Health.

[2] Yellowlees P. (2005). Successfully developing a telemedicine system. J. Telemed. Telecare.

[3] Broens T.H., Vollenbroek-Hutten M.M., Hermens H.J., van Halteren A.T., Nieuwenhuis L.J. (2007). Determinants of successful telemedicine implementations: A literature study. J. Telemed. Telecare.

[4] South African Government eHealth Strategy for South Africa, 2012–2016. http://www.doh.gov.za/docs/stratdocs/2012.

[5] Handler T.J. (2007). Hype cycle for telemedicine. Gart. Ind. Res..

[6] Sood S., Mbarika V., Jugoo S., Dookhy R., Doarn C.R., Prakash N. (2007). What is telemedicine? A collection of 104 peer-reviewed perspectives and theoretical underpinnings. Telemed. e-Health.

[7] Ekeland A.G., Bowes A., Flottorp S. (2011). Methodologies for assessing telemedicine: A systematic review of reviews. Int. J. Med. Inform..

[8] Van der Wetering R., Batenburg R. (1997). A framework for the economic evaluation of telemedicine. J. Telemed. Telecare.

[9] Ethal E. Sustainable Telemedicine: Paradigms for Future-Proof Healthcare. http://armtelemed.org/resources/48-EHTEL_BriefingPaperSustainableTelemedicine.pdf.

[10] Kidholm K., Ekeland A.G., Jensen L.K., Rasmussen J., Pedersen C.D., Bowes A., Flottorp S.A., Bech M. (2012). A model for assessment of telemedicine applications: MAST. Int. J. Technol. Assess. Health Care.

[11] Hicks L.L., Boles K.E. (2004). A comprehensive model for evaluating telemedicine. Stud. Health Technol. Inform..

[12] Attewell P. (1992). Technology diffusion and organizational learning: The case of business computing. Organ. Sci..

[13] Khoja S., Scott R., Mohsin M., Ishaq A.F.M., Casebeer A.L. (2006). Developing a conceptual-framework for e-health readiness assessment tools for developing countries. ICT Develop..

[14] Cilliers L., Flowerday S.V. (2013). Health information systems to improve health care: A telemedicine case study. SA J. Inf. Manag..

[15] Alikarami R., Moghadam R.A., Javadi S.R.S., Vahdat D. (2011). Evaluation of effecting factors on success of telemedicine systems (using models of TAM and UTAUT). Can. J. Netw. Inf. Secur..

[16] Khoja S., Scott R., Casebeer A., Mohsin M., Ishaq A., Gilani S. (2007). E-health readiness assessment tools for healthcare institutions in developing countries. Telemed. e-Health.

[17] Buchachi F., Pakenham-Walsh N. (2007). Information technology of health in developing countries. Chest.

[18] Pelletier-Fleury N., Fargeon V., Lanoé J., Fardeau M. (1997). Transaction costs economics as a conceptual framework for the analysis of barriers to the diffusion of telemedicine. Health Policy.

[19] Tanriverdi H., Iacono C.S. Knowledge Barriers to Diffusion of Telemedicine. Proceedings of the International Conference of the Association for Information Systems.

[20] Bashshur R., Shannon G., Krupinski E., Grigsby J. (2011). The taxonomy of telemedicine. Telemed. e-Health.

[21] Telehealth and Telecare Aware. What is Telecare?. http://telecareaware.com.

[22] Grigsby J., Rigby M., Hiemstra A., House M., Olsson S., Whitten P. (2002). The diffusion of telemedicine. Telemed. J. e-Health.

[23] Scott W.R. (1990). Innovation in medical care organizations: A synthetic review. Med. Care Res. Rev..

[24] Jennett P., Gagnon M., Brandstadt H. (2005). Preparing for success: Readiness models for rural telehealth. J. Postgrad. Med..

[25] Legare E., Vincent C., Lehoux P., Anderson D., Kairy D., Gagnon M. (2010). Telehealth readiness assessment tools. J. Telemed. Telecare.

[26] Khoja S., Durrani H., Scott R., Sajwani A., Piryani U. (2013). Conceptual framework for development of comprehensive e-Health evaluation tool. Telemed. e-Health.

[27] Chattopadhyay S., Li J., Land L., Ray P. A Framework for Assessing ICT Preparedness for e-Health Implementations. Proceedings of the 10th International Conference on e-Health Networking, Applications and Services.

[28] Tamburis O., Mangia M., Contenti M., Mercurio G., Mori A.R. (2012). The LITIS conceptual framework: Measuring eHealth readiness and adoption dynamics across the Healthcare Organizations. Health Technol..

[29] Leon N., Schneider H., Daviaud E. (2012). Applying a framework for assessing the health system challenges to scaling up mHealth in South Africa. BMC Med. Inform. Decis. Mak..

[30] Chipps J., Mars M. (2012). Readiness of healthcare institutions in Kwazulu-Natal to implement telepsychiatry. J. Telemed. Telecare.

[31] Durrani H., Khoja S. (2012). Health needs and eHealth readiness assessment of health care organizations in Kabul and Bamyan, Afghanistan. EMHJ.

[32] Venkatesh V., Morris M.G., Davis G.B., Davis F.D. (2003). User acceptance of information technology: Toward a unified view. MIS Q..

[33] Dünnebeil S., Sunyaev A., Blohm I., Leimeister J.M., Krcmar H. (2012). Determinants of physicians technology acceptance for e-health in ambulatory care. Int. J. Med. Inform..

[34] Finch T., Mair F., May C. (2006). Teledermatology in the UK: Lessons in service innovation. Br. J. Dermatol..

[35] Van Dyk L. (2013). The Development of a Telemedicine Maturity Model. Ph.D. Dissertation.

